# Editorial on the Special Issue “Advances in Composite Gels”

**DOI:** 10.3390/gels9010046

**Published:** 2023-01-06

**Authors:** Hiroyuki Takeno

**Affiliations:** 1 Division of Molecular Science, Graduate School of Science and Technology, Gunma University, Kiryu 376-8515, Gunma, Japan; takeno@gunma-u.ac.jp; 2 Gunma University Center for Food Science and Wellness, 4-2 Aramaki, Maebashi 371-8510, Gunma, Japan

Polymer gels are soft materials composed of a large amount of solvent (water, organic solvent, and ionic liquid) and a polymer, and they are constructed using a three-dimensional network. The incorporation of other components into polymer gels gives birth to functional materials that cannot be attained in gels constituted of a single polymer. For example, polymer gels produced through conventional methods are mechanically brittle; by contrast, composite gels successfully incorporate inorganic nanoparticles and nanofibers into the polymer network, thus acquiring mechanically tough characteristics. Moreover, polymer composite hydrogels have potential applications in various fields such as tissue engineering, biomedical engineering, electrochemistry, and environmental chemistry. To fabricate smart composite gels, it is necessary to control the structural morphology of composite gels, and the interactions between the additive and the polymer network [1,2].

In addition to polymer composite gels, supramolecular gels formed via the self-assembly of small-molecular gelators and surfactants have raised much interest in terms of the production of smart nanomaterials [3]. A self-assembling structure is formed through non-covalent bonds such as hydrogen bonds, electrostatic interactions, π−π interactions, and van der Waals interactions. Factors such as molecular interactions, molecular architecture, and chirality significantly affect the self-assembling morphology. Recent studies highlight the importance of controlling the dimension of the supramolecular systems [4] and complex-ordered aggregates that cause aggregation-induced emission [5]. Moreover, two-component gelator systems including the combination of polymers and surfactants are also promising in the fabrication of smart nanomaterials [4].

In recent years, many advances have been made in the development of novel composite gels. This book aims to present the latest findings of composite gels by experts around the world in various fields. Each chapter in this book has been previously published in a Special Issue of the international journal, *Gels*, entitled “*Advances in Composite Gels*”. These articles reveal the promising potential of composite gels as materials for cell scaffolds [6,7], opt-electrical devices [8], energy storage devices [9], catalysts [8], biomedicine [10], drug delivery systems [11], protein quantification [12], dental mold gypsum [13], excellent photoluminescence properties [5], and wastewater remediation [14]. Recent advances in composite hydrogels deal with biomaterials [15], as well as bioinspired and biomimetic materials [7]. Learning nature’s strategy to efficiently fabricate smart materials proves valuable. The extracellular matrix is a three-dimensional network composed of macromolecular systems [7], which provide an adequate environment for cell adhesion, cell proliferation, and cell differentiation [6]. Furthermore, the eye lens having adaptive and focus-tunable characteristics may be regarded as a kind of biological gel [16]. Gel polymer electrolytes garner much attention due to having electrochemical applications such as portable electro devices and soft gel actuators [9,17]. The use of two-component additives produces composite gels with multi-functionality and excellent material properties, possibly resulting in a synergistic effect between the additives [2,18]. The incorporation of inorganic nanoparticles and nanofibers into a gel matrix provides robust and highly stretchable composite gels constructed by multi-crosslinking [2]. Additionally, the review articles included in this collection introduce recent advances in drug delivery systems [19,20], microgels for oil recovery [21], and multi-layer hydrogels [22]. 

Finally, I would like to express my sincere gratitude to all the contributing authors who have made great contributions to the publication of this book. 

## References

[1] Nagai D., Isobe N., Inoue T., Okamoto S., Maki Y., Yamanobe T. (2022). Preparation of Various Nanomaterials via Controlled Gelation of a Hydrophilic Polymer Bearing Metal-Coordination Units with Metal Ions. Gels.

[2] Takeno H., Suto N. (2021). Robust and Highly Stretchable Chitosan Nanofiber/Alumina-Coated Silica/Carboxylated Poly (Vinyl Alcohol)/Borax Composite Hydrogels Constructed by Multiple Crosslinking. Gels.

[3] Azum N., Rub M.A., Khan A., Alotaibi M.M., Asiri A.M. (2022). Synergistic Interaction and Binding Efficiency of Tetracaine Hydrochloride (Anesthetic Drug) with Anionic Surfactants in the Presence of NaCl Solution Using Surface Tension and UV–Visible Spectroscopic Methods. Gels.

[4] Zhang P., Liu Y., Fang X., Ma L., Wang Y., Ji L. (2022). Stoichiometric Ratio Controlled Dimension Transition and Supramolecular Chirality Enhancement in a Two-Component Assembly System. Gels.

[5] Liu X., Li C., Wang Z., Zhang N., Feng N., Wang W., Xin X. (2021). Luminescent Hydrogel Based on Silver Nanocluster/Malic Acid and Its Composite Film for Highly Sensitive Detection of Fe3+. Gels.

[6] Vitale M., Ligorio C., Smith I.P., Richardson S.M., Hoyland J.A., Bella J. (2022). Incorporation of Natural and Recombinant Collagen Proteins within Fmoc-Based Self-Assembling Peptide Hydrogels. Gels.

[7] Riacci L., Sorriento A., Ricotti L. (2021). Genipin-Based Crosslinking of Jellyfish Collagen 3D Hydrogels. Gels.

[8] Özuğur Uysal B., Nayır Ş., Açba M., Çıtır B., Durmaz S., Koçoğlu Ş., Yıldız E., Pekcan Ö. (2022). 2D Materials (WS2, MoS2, MoSe2) Enhanced Polyacrylamide Gels for Multifunctional Applications. Gels.

[9] Velez A.A.I., Reyes E., Diaz-Barrios A., Santos F., Fernández Romero A.J., Tafur J.P. (2021). Properties of the PVA-VAVTD KOH Blend as a Gel Polymer Electrolyte for Zinc Batteries. Gels.

[10] Ekama S.O., Ilomuanya M.O., Azubuike C.P., Ayorinde J.B., Ezechi O.C., Igwilo C.I., Salako B.L. (2021). Enzyme Responsive Vaginal Microbicide Gels Containing Maraviroc and Tenofovir Microspheres Designed for Acid Phosphatase-Triggered Release for Pre-Exposure Prophylaxis of HIV-1: A Comparative Analysis of a Bigel and Thermosensitive Gel. Gels.

[11] Rub M.A., Azum N., Kumar D., Asiri A.M. (2022). Interaction of TX-100 and Antidepressant Imipramine Hydrochloride Drug Mixture: Surface Tension, 1H NMR, and FT-IR Investigation. Gels.

[12] Helbing D.L., Böhm L., Oraha N., Stabenow L.K., Cui Y. (2022). A Ponceau S Staining-Based Dot Blot Assay for Rapid Protein Quantification of Biological Samples. Gels.

[13] Ma L., Xie Q., Evelina A., Long W., Ma C., Zhou F., Cha R. (2021). The Effect of Different Additives on the Hydration and Gelation Properties of Composite Dental Gypsum. Gels.

[14] Sharma G., Kumar A., Ghfar A.A., García-Peñas A., Naushad M., Stadler F.J. (2021). Fabrication and Characterization of Xanthan Gum-cl-poly(acrylamide-co-alginic acid) Hydrogel for Adsorption of Cadmium Ions from Aqueous Medium. Gels.

[15] Tunit P., Thammarat P., Okonogi S., Chittasupho C. (2022). Hydrogel Containing Borassus flabellifer L. Male Flower Extract for Antioxidant, Antimicrobial, and Anti-Inflammatory Activity. Gels.

[16] Malik A., Khan J.M., Alhomida A.S., Ola M.S. (2022). Modulation of the Structure and Stability of Novel Camel Lens Alpha-Crystallin by pH and Thermal Stress. Gels.

[17] Sadiq N.M., Aziz S.B., Kadir M.F.Z. (2022). Development of Flexible Plasticized Ion Conducting Polymer Blend Electrolytes Based on Polyvinyl Alcohol (PVA): Chitosan (CS) with High Ion Transport Parameters Close to Gel Based Electrolytes. Gels.

[18] Safri A., Fletcher A.J. (2022). Effective Carbon/TiO2 Gel for Enhanced Adsorption and Demonstrable Visible Light Driven Photocatalytic Performance. Gels.

[19] Xie Y., Guan Q., Guo J., Chen Y., Yin Y., Han X. (2022). Hydrogels for Exosome Delivery in Biomedical Applications. Gels.

[20] Sastri T.K., Gupta V.N., Chakraborty S., Madhusudhan S., Kumar H., Chand P., Jain V., Veeranna B., Gowda D.V. (2022). Novel Gels: An Emerging Approach for Delivering of Therapeutic Molecules and Recent Trends. Gels.

[21] Rozhkova Y.A., Burin D.A., Galkin S.V., Yang H. (2022). Review of Microgels for Enhanced Oil Recovery: Properties and Cases of Application. Gels.

[22] Jin L., Xu J., Xue Y., Zhang X., Feng M., Wang C., Yao W., Wang J., He M. (2021). Research Progress in the Multilayer Hydrogels. Gels.

